# 3D laparoscopic treatment of bladder cancer with pelvic multi-organ invasion: a case report and literature review

**DOI:** 10.3389/fonc.2023.1249389

**Published:** 2023-10-18

**Authors:** Zheng Chen, Kaifeng Deng, Luping Sun, Lijun Qu, Xinhui Chao, Jingmin Rao, Caimmei Hong, Yumin Zhuo, Zhichao Lin, Caiyong Lai

**Affiliations:** ^1^ Department of Urology, The First Affiliated Hospital of Jinan University, Guangzhou Guangdong, China; ^2^ Medical Image Center, The First Affiliated Hospital of Jinan University, Guangzhou Guangdong, China

**Keywords:** radical cystectomy, pelvic lymph node dissection, ileal conduit, muscle-invasive bladder cancer, 3D laparoscopy-assisted treatment

## Abstract

**Introduction:**

Radical cystectomy with dissection of pelvic lymph nodes and urethral diversion is the standard surgical treatment for muscle-invasive non-metastatic bladder cancer. In rare cases where patients with bladder cancer without distant metastasis have pelvic multi-organ invasion, the cancer compresses or invades the ureter and, in severe cases, leads to bilateral upper urinary tract obstruction and renal damage. The treatment recommended by guidelines often cannot improve the patients’ clinical symptoms immediately, and patients cannot complete the treatment owing to severe side effects, resulting in poor survival benefits.

**Case presentation:**

A 69-year-old woman with facial edema was treated at the First Affiliated Hospital of Jinan University. The serum creatinine and potassium values were 1244 umol/L and 5.86 mmol/L, respectively. Pelvic magnetic resonance and abdominal computed tomography revealed that the bladder tumor had infiltrated the uterus, anterior vaginal wall, rectum, right ureter, right fallopian tube, and right ovary and metastasized to multiple pelvic lymph nodes. Tumor invasion of the right ureter resulted in severe hydronephrosis of the right kidney and loss of function and obstructive symptoms in the left kidney. Four days later, the patient’s creatinine level decreased to 98 u mol/L, the general condition significantly improved, and the patient and family members strongly desired surgical treatment of the tumor. Through a comprehensive preoperative discussion, possible intraoperative and postoperative complications were evaluated. Right nephrectomy, right ureterectomy, total pelvic organ resection, extended pelvic lymph node dissection, and bowel and urinary diversion were conducted under 3D laparoscopy-assisted treatment. The patient was followed-up for 1.5 years and showed good tumor control, self-care, and mental status.

**Conclusion:**

Minimally invasive surgery is a curative option for patients with bladder cancer with pelvic multi-organ invasion without distant metastasis. Surgeons should strictly control the indications for surgery and warn patients about the occurrence of related post-surgical complications.

## 1 Introduction

Bladder cancer has a high incidence and mortality (1). More than 25% of newly diagnosed patients with bladder cancer in China have muscle-invasive bladder cancer (MIBC); of these, 20–30% of the MIBC cases occur following treatment (2), and the prognosis is poor. Surgery remains the primary treatment for invasive bladder cancer (3). A bladder tumor with flat outward growth is often a sign of late-stage disease and is commonly accompanied by infiltration of the surrounding organs and ureters, causing serious renal function damage (4). Surgical procedures for bladder cancer without distant metastasis complicated by multi-organ pelvic infiltration are not recommended by the guidelines because of the excessive difficulty and postoperative complications. Currently, the treatments of advanced cases of bladder cancer focus on various types of radiotherapy, chemotherapy, or combined immunotherapy (5); minimally invasive surgical treatment is rarely performed.

From a large sample database research, the results of the patients with MIBC who underwent conservative treatment had short survival time in surgery (6, 7). Some researchers believe that conservative treatment prolongs radical cystectomy and may delay the treatment of patients who do not respond to chemotherapy (8, 9). In addition, the toxicity and side effects of radiotherapy and chemotherapy are relatively high, which influence the adhesion of organs and subsequent selection of urine diversion procedures. Regarding treatment costs, studies have shown that conservative treatment is often accompanied by a high economic burden (10). Therefore, conservative treatment and its side effects often cannot immediately improve the clinical symptoms of patients, and some patients cannot complete the treatment owing to the side effects or economic burden, therefore decreasing the survival benefit.

Traditional open radical resection of bladder cancer has limited visual field exposure and induces a large surgical incision, increased bleeding, increased trauma to patients, relatively higher postoperative pain, gastrointestinal function recovery, and other complications. Laparoscopic radical nephrectomy was first performed by Clayman in 1991 (11). Parra first reported laparoscopic radical cystectomy in 1992 (12). With the continuous progress in minimally invasive technology, robot-assisted laparoscopic radical cystectomies have become mainstream. Compared with open surgery, laparoscopic surgery induces less trauma and faster recovery and can significantly enhance postoperative recovery and patients’ quality of life. Regarding laparoscopic radical nephroureterectomy, Petrut have reported three cases (13), Tanaka have reported two (14), and Słojewski have reported one (15), all of which revealed that the operation was safe. Therefore, we believe that simultaneous laparoscopic radical nephroureterectomy and radical cystectomy is a safe, feasible, and effective method for radical cystectomy in bladder carcinoma. We found that in the existing reports, a two-dimensional laparoscopic technique was used, which lacked three-dimensional (3D) sensation, low intraoperative accuracy, and a high risk of bleeding. No reports have shown that the 3D laparoscopic technology can be used to perform nephroureterectomy, ureterectomy, total pelvic viscerectomy, extended lymph node dissection, or urinary diversion. We successfully applied this technique to the surgical treatment of a patient diagnosed with bladder cancer without distant metastasis but with pelvic multi-organ invasion. Combined with clinical practice, related literature was reviewed regarding surgeries, postoperative complications, and treatment options. We conducted a comprehensive analysis of the feasibility of the proposed method.

## 2 Case description

**Figure 1:**
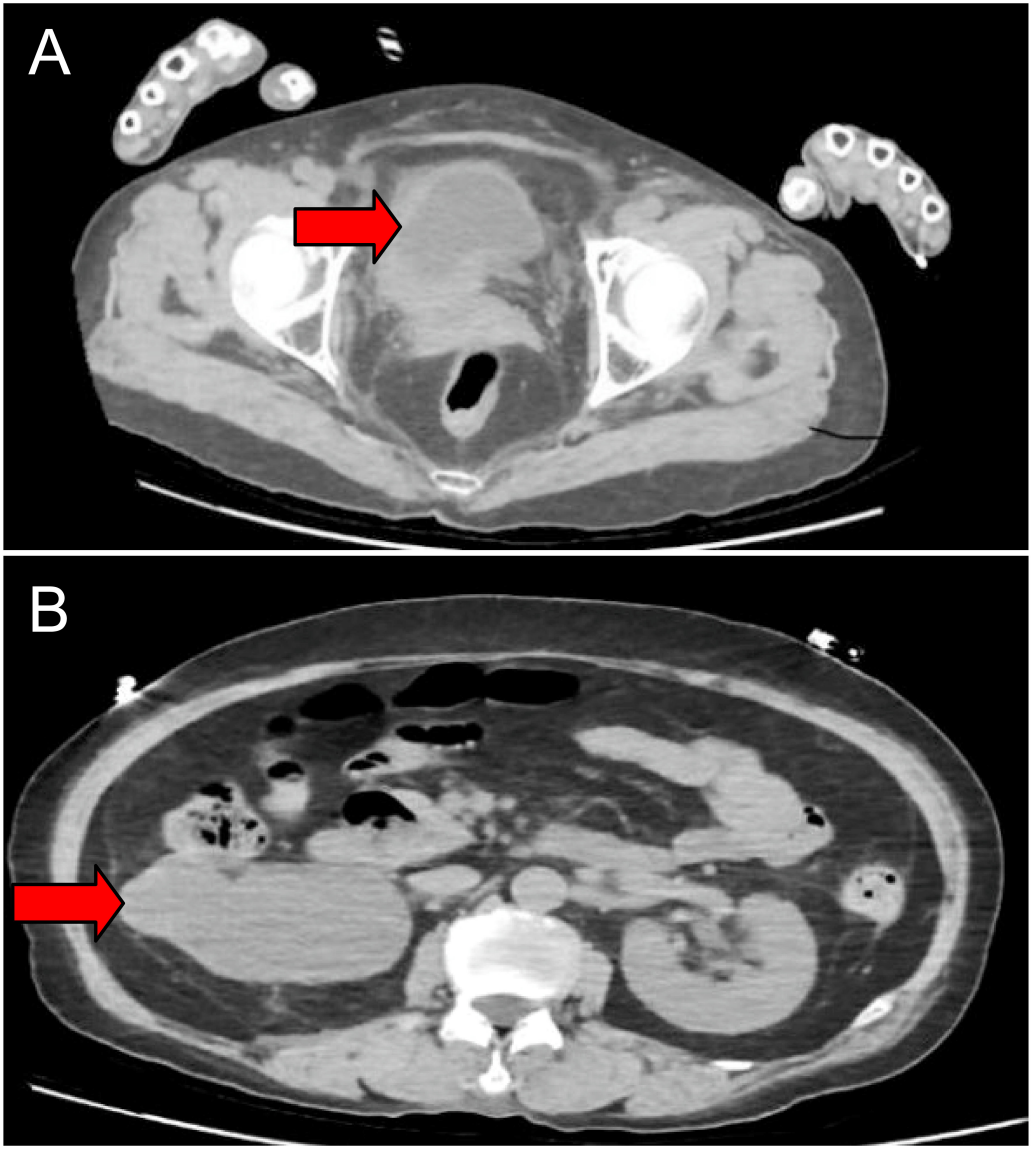
Imaging studies before treatment. CT showing thickening in the right lateral wall of the urinary bladder (cross-sectional) **(A)**. Severe right hydronephrosis(cross-sectional) **(B)**.

**Figure 2:**
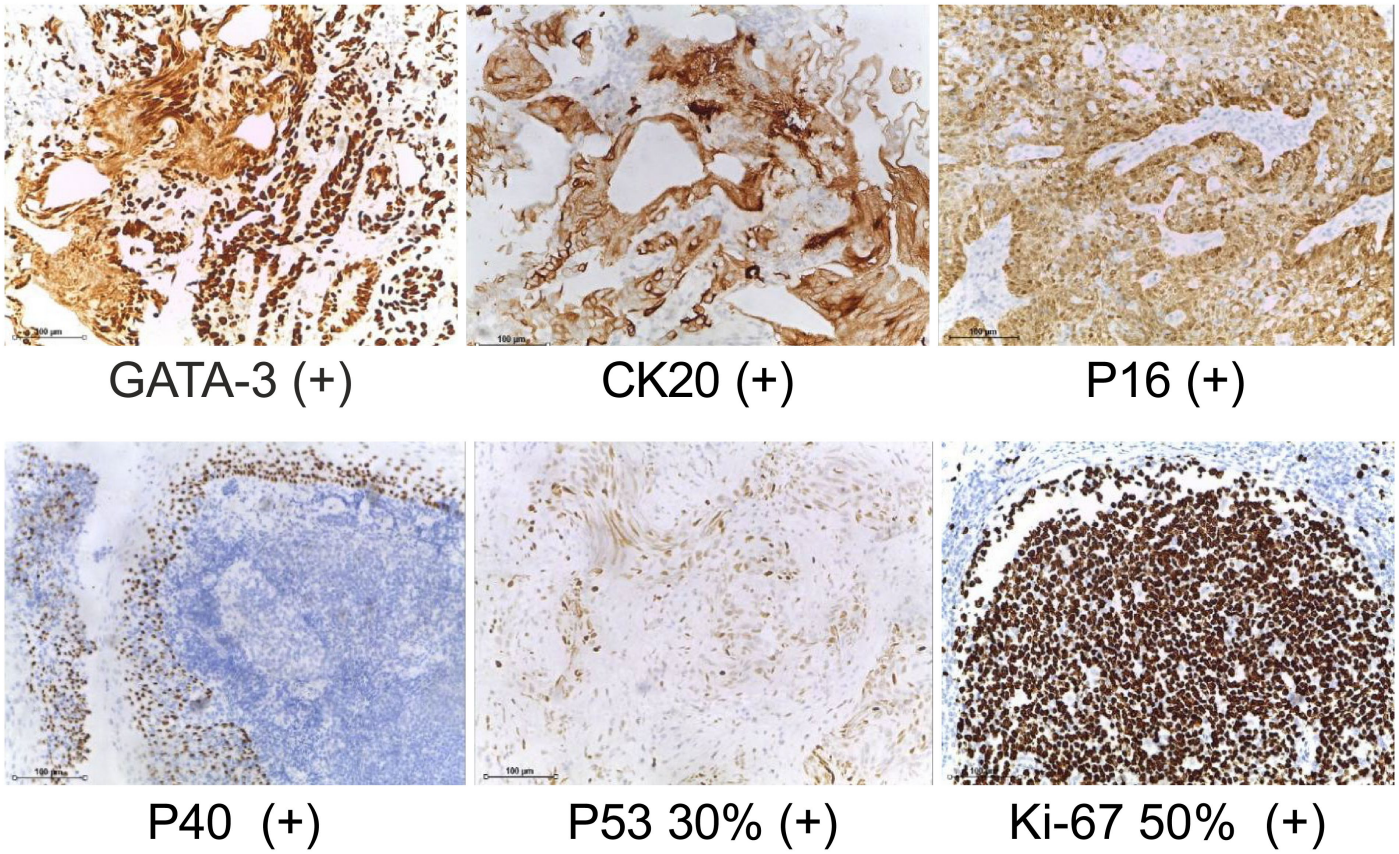
Bladder pathological biopsy. (IHC staining, ×40) GATA3, CK20, P16, P40, P53 and Ki-67 are expressed.

**Figure 3:**
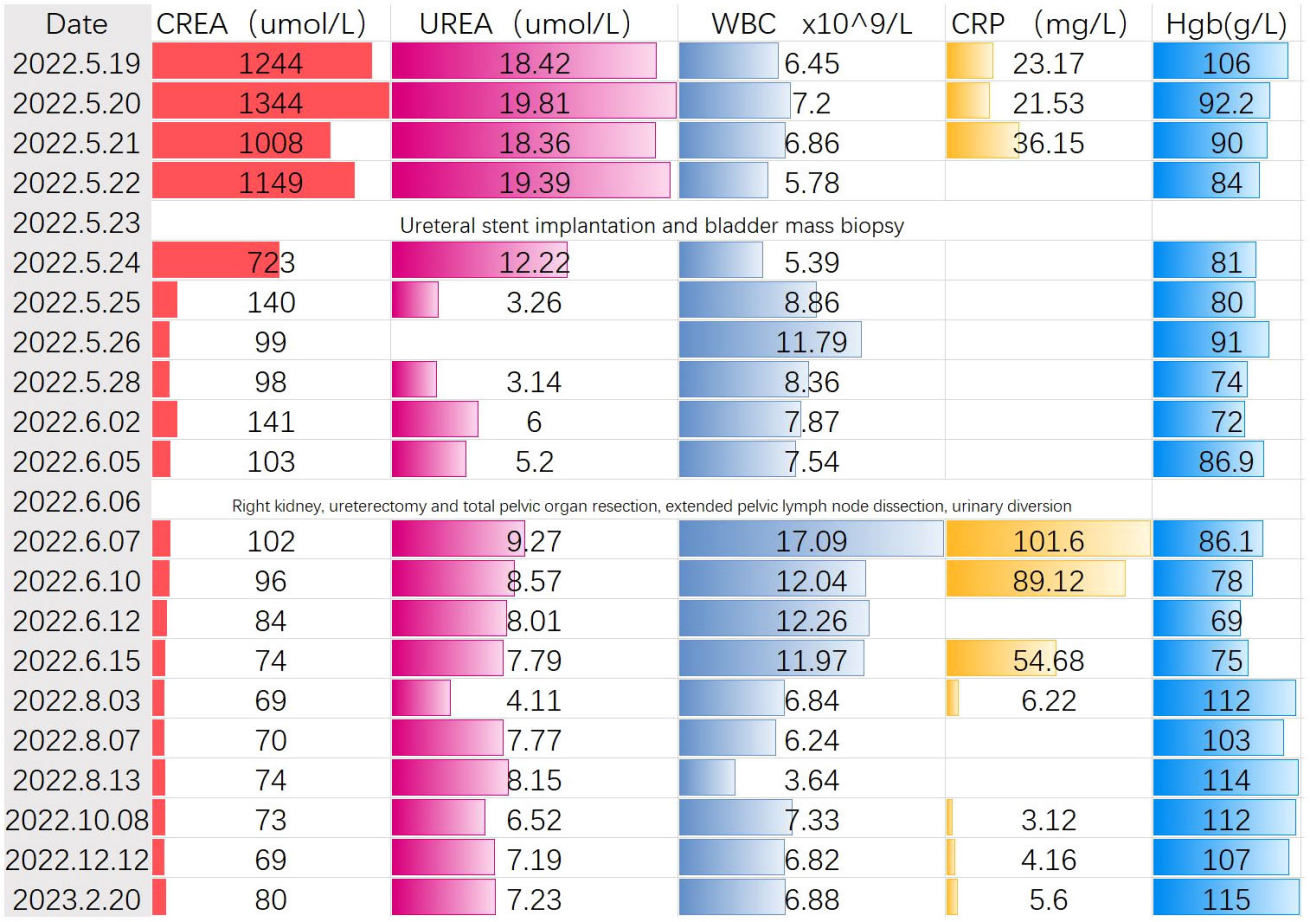
Changes in preoperative and postoperative examination indexes of patients.

We present the case of a 69-year-old woman who initially presented with facial edema, with no chills, fever, nausea, vomiting, or irritation. No significant findings were noted for previous medical behaviors, medication, social, or family histories. The local hospital detected creatinine (CREA) of 1244 umol/L, blood potassium of 5.86 mmol/L, and urea (UREA) of 18.42 umol/L. Imaging revealed that the tumor had invaded the uterus, anterior vaginal wall, rectum, right ureter, right fallopian tube, and right ovary, and multiple pelvic lymph node metastases were observed. The tumor invasion of the right ureter caused severe hydronephrosis of the right kidney and loss of function and mild hydronephrosis of the left kidney (**Figure 1**). In this patient, we used a whole-digestive tract lavage with polyethylene glycol electrolyte solution for mechanical bowel preparation, and cephalosporin was used preoperatively according to the patient’s drug sensitivity test for antibiotic bowel preparation.We first performed a left ureteral stent placement and bladder mass biopsy. A postoperative pathological biopsy of the bladder mass suggested an invasive urothelial carcinoma (**Figure 2**). Creatinine levels significantly decreased following surgery (**Figure 3**), and 4 days following ureteral stent placement, the creatinine was 98 umol/L. Patients and their families strongly desired surgical treatment. After a multidisciplinary consultation, we decided to perform unilateral nephroureterectomy, total pelvic exenteration, extended pelvic lymph node dissection, and small and large bowel diversion under 3D laparoscopy.

**Figure 4:**
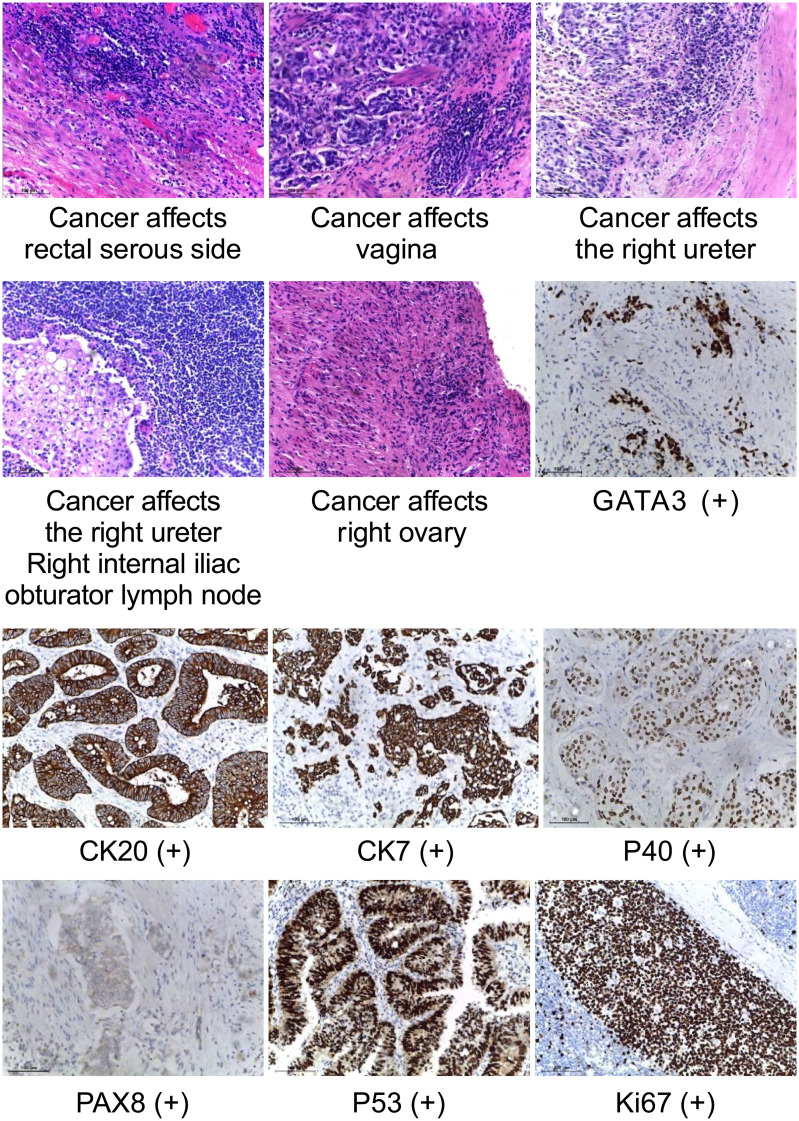
Histopathology of surgically resected tumors. (HE staining, ×40)visible tumor cells in the serous side of rectum, vagina, right ureter, right ovary, and right internal iliac obturator lymph node. (IHC staining, ×40) GATA 3, CK20, CK7, P40, PAX8,P53 and Ki-67 are expressed.

We used 3D laparoscopy to perform nephrectomy using the extraperitoneal approach. Following the removal of the right kidney, the right ureter was separated to the level of the iliac vessels. Then, the left ureter was separated using the peritoneal approach, and bilateral ureteral lymph node dissection was conducted. The bilateral internal and external iliacus, obturator, and common iliacus lymph nodes were dissected. Considering that the tumor invaded the uterine adnexa and rectum, we decided to perform a radical pelvic organ resection of the uterus, bilateral adnexa, vagina, and rectum. A suspicious positive resection of the distal rectal margin was performed because of intraoperative findings of tumor invasion in the anterior wall of the vagina and part of the urethra (**Figure 4**). Vaginal and rectal lysis, ileal conduit placement, and sigmoid colostomy were performed.

The left ureter was fully mobilized. An anastomosis was made at the ileal conduit, whereas the other end was connected to the right abdominal stoma. An incision was made at the lateral margin of the rectus abdominis in the left lower abdomen. The proximal intra-abdominal mesentery of the sigmoid colon was fixed to the peritoneum of the left-side abdominal wall, and the stump was bandaged and sutured layer-by-layer. The operative time was 510 min, and blood loss was approximately 100 ml. No intra-or postoperative complications were observed. Postoperative progress was uneventful, and the patient was discharged home on postoperative day 20. Postoperative follow-up examination revealed that the patient healed well without progression on imaging studies and was in good spirits.

## 3 Discussion

In recent years, some high-quality randomized controlled trials have shown that, compared with 2D laparoscopy, the application of 3D laparoscopy in basic operation training may shorten the duration of operation, reduce operation errors, and improve operation accuracy (16–19). Some researchers have studied the use of 2D and 3D laparoscopic techniques, such as grasping, cutting, suturing, and knotting, to simulate the basic operations of surgery. Compared to 2D laparoscopy, 3D laparoscopy achieves a 36% faster completion of the same procedure and a 62% lower error rate (20). The 3D laparoscopic system has a high-definition 3D surgical field of view, and its price is not significantly higher than that of traditional 2D laparoscopic systems. Therefore, it is more conducive for promotion and application.

During the perioperative period, we organized a preoperative discussion on possible complications related to the patient. Infectious complications are common following cystectomy, and the incidence of infection post-cystectomy can reach 25%. A study of the National Surgical Quality Improvement Program database has found that nearly half of the readmissions were because of infectious causes (21). Notably, >50% of the complications occurred following discharge, most commonly at the end of week 2. Postoperative wound infections included fever, abscesses, urinary tract infection, sepsis, and pyelonephritis (22). Parker et al. have found an association between diabetes mellitus (OR, 2.27) and the rate of perioperative blood transfusions (OR, 1.58) and postoperative urinary tract infections in a large group of patients undergoing radical cystectomy (23). Another study has found that an operation time ≥480 min was associated with postoperative urinary tract infection (24). Some researchers have found an independent association between the presence of hydronephrosis and development of urinary tract infections (OR, 4.2; 95% CI, 1.525–11.569; P=0.006) (25). In other related studies, body mass index and ureteral strictures have been reported as potential risk factors for urinary tract infections (26). In contrast, Mano et al. have noted that age, sex, diabetes, perioperative chemotherapy, and the bowel segment used for reconstruction were not associated with urinary tract infection (21). Owing to the inconsistency in the results of the risk factor analysis in the above studies, we believe that further prospective studies on factors that cause infection should be conducted. In this case, we prescribed preoperative cephalosporin antibiotics based on urine culture results. Therefore, pre-operative screening can provide appropriate preventive measures. A study at Indiana University revealed that, for patients who tested positive for *Clostridium difficile* pre-surgery, immediate treatment with protective isolation and metronidazole reduced the rate of symptomatic infection post-surgery by about half (27).

Urogenital complications (renal insufficiency, urinary leakage, ureteral tract obstruction/stenosis, deterioration of renal function in the long run, and electrolyte disturbances) often occur because the normal urinary flow path is altered. With studies indicating an incidence of 10–30% (28). This is because of perioperative fluid loss and fluid shift. Current critical concepts in rapid rehabilitation surgery include optimizing intra-operative fluid management to prevent fluid overload. Specific approaches to fluid therapy vary and include colloid administration, restrictive fluid administration, administration of fluids for specific hemodynamic parameters, and the use of vasopressors to maintain arterial pressure. Regardless of the approach, efforts have been made to minimize intravenous fluids. In a retrospective review of the administration of restrictive fluids and vasopressors during cystectomy predicted postoperative AKI (29). In many cases, AKI resolves after liquid resuscitation. Thus, the patient’s clinical fluid status and cardiac comorbidities should be taken notice to avoid fluid overload. If renal impairment persists or progresses after adequate fluid resuscitation, renal ultrasound imaging should be considered to assess hydronephrosis and rule out urinary obstruction.

At present, there are three main methods of urinary diversion, including orthotopic neobladder, ileal conduit and cutaneous ureterostomy. Cutaneous ureterostomy is a simple and safe procedure. It is suitable for patients with short life expectancy, intestinal disorders that cannot use the bowel for urinary diversion, or general conditions that cannot tolerate surgery. Due to the small diameter of the ureter, the probability of cutaneous stoma stenosis is very high. Orthotopic neobladder has the advantage of not requiring abdominal stoma, which is beneficial to maintain their own image. However, there are many contraindications, such as patients with high-dose preoperative radiotherapy, patients with unresectable tumor, patients with high possibility of postoperative pelvic local recurrence, and female patients with tumor invasion of bladder neck and anterior vaginal wall. Some patients with orthotopic neobladder need long-term catheterization or intermittent self-catheterization, which frequently causes urinary tract infections. At the same time, the incidence of postoperative hydronephrosis is higher than the other two urinary diversion methods. This may be related to the need for more wound anastomosis, including the anastomosis between the bilateral ureteral stumps and the neobladder and the anastomosis between the neobladder and the urethra. Relevant studies have shown that the incidence of hydronephrosis in patients after urinary diversion is about 11.4%, and about 12.8% of them are due to tumor recurrence. It also cautions that if a patient has postoperative hydronephrosis, imaging or further ureteroscopy should be performed to rule out malignant strictures. Ileal conduit surgery is one of the classic and most commonly used urinary diversion procedures. In recent years, the development of modified ileal conduit surgery has significantly reduced the occurrence of related complications. The common complications of ileal conduit surgery focus on ureteroileal anastomotic leakage or stenosis and stoma-related complications. The early clinical manifestations of ureteroileal anastomotic stenosis are insidious, and it is often noticed when the patients have recurrent fever or lumbago. Therefore, we recommend that the patients should regularly review the urinary system color Doppler ultrasound after surgery, and IVU examination should be performed if necessary to determine the urinary obstruction. In this case, we chose ileal conduit after fully communicating with the patient and his family, and comprehensively evaluating various urethral diversion methods.

In the present case, we performed diversion of the fecal-urine shunt, which involved the use of two stomas. This allowed free drainage of urine without fecal contact. The risk of ascending pyelonephritis is significantly reduced by completely separating the fecal contents to prevent stool reflux into the ureteral anastomosis. However, urethral diversion carries the risk of complications, such as ureteral anastomotic stenosis, ureteral stricture, and stoma hernia. These complications can lead to irreversible kidney damage and other infection-related complications. Secondary surgery may require complex reconstruction and may cause additional losses.

Ureteroileal anastomotic stenosis may be caused by poor surgical techniques for ureteral anastomosis or ischemic injury following ureteral mobilization. To reduce this risk, we checked the correct position of the mesentery to prevent angulation and over-compression, which could lead to ischemia during ileal neovascularization. The left distal portion of the ureter was removed to achieve a tension-free ureterostomy without angulation. The papilla formed in the body after the ileal segment was cut. The ileal catheter was led out of the abdominal via an extraperitoneal tunnel and was secured to the aponeuroses of the external oblique and transverse abdominis muscles. To make the ureteral anastomoses, the tip of the ureter was divided to form a large opening and anastomosed end-to-side to the ileum with continuous strangulated suture. In most instances, ureteric strictures are either secondary to ureteral ischemia or periureteral fibrosis and develop less than 1–2 years following surgery. Ureteral obstruction is usually asymptomatic and is often incidentally discovered during follow-up imaging or laboratory tests (30–32). Ureteral stenosis can be a consequence of ischemia; therefore, it is critical to minimize ureteric devascularization during the procedure and directly manipulate the ureter to the greatest extent possible, which will restrict the ureteric outer membrane sheath surrounding the blood supply of small artery injury.

The effect of stoma hernia on quality of life and efforts to reduce its occurrence are of great significance. An Indiana University study among patients with ileal conduit disease has reported an overall morbidity rate of 29% and a surgical repair rate of 45%. The most common symptoms were abdominal discomfort (58%), intestinal obstruction or strangulation (15%), and partial small bowel obstruction (15%) (33). The rectus abdominis plays a role in maintaining abdominal pressure and assists in respiration and defecation. It works together with the inner oblique abdominal, transversus abdominis, and outer oblique abdominis muscles to protect abdominal organs and maintain organ stability.

Finally, there is a risk of venous thromboembolic disease (VTE) factor for all major surgical interventions; however, the additional risk increases when malignant tumors are present (34). An analysis has revealed that the incidence of VTE complications post-cystectomy ranged from 3% to 11.6% (35). Preventive measures for VTE following radical cystectomy are crucial. Among the prophylactics, heparin is recommended. Postoperative challenges will arise regarding the timing of the assessment of anticoagulation therapy re-initiation, and the risk of further VTE, compared to postoperative bleeding, will remain vigilant.

Cisplatin-based neoadjuvant chemotherapy(NAC), gemcitabine plus cisplatin (GC), and the dose-dense methotrexate–vinblastine (ddMVAC) are the recommended standard treatments for MIBC. The disadvantages of NAC include its dependence on the delays or progression related to clinical stage and treatment, which may affect deterministic and curative therapies. Chemotherapy may include cisplatin-based regimens; however, approximately 50% of the patients with MIBC are clinically not eligible for cisplatin chemotherapy owing to age and/or disease-related risk factors (36). Carboplatin can be considered in patients with kidney insufficiency; however, its effectiveness is not as satisfactory as that of cisplatin (37). Patients who relapse or progress after first-line therapy have a poor prognosis. Second-line chemotherapy regimens, with an objective response rate (ORR) of 12% and a median overall survival (mOS) of 5–7 months (38), have been used in a variety of settings. Related studies have shown that NAC performed 8 weeks after the diagnosis of MIBC increases the risk of advanced tumor and positive lymph node. When the time between the end of NAC and cystectomy is more than 12 weeks, the risk of lymph node invasion significantly increases. Some studies have shown that the longer the time between NAC and radical cystectomy, the faster the tumor progresses, but the optimal interval between NAC and radical cystectomy still needs further study. At the same time, researchers believe that adjuvant chemotherapy may affect the choice of organ adhesion and urethral shunting. Some patients often cannot complete the treatment due to side effects or financial burden, thus reducing the survival benefit. The patient and her family members all refused the scheme of preoperative adjuvant chemotherapy, so we did not perform adjuvant chemotherapy in this clinical case.

With the application and exploration of immunotherapy gradually covering the advanced stage to the locally advanced stage, or even the earlier stage of the disease, immunotherapy with PD-1 or PD–LI inhibitors is expected to change the conditions of bladder cancer treatment. In the neoadjuvant therapy stage, the tumor load is high, and the efficacy of neoadjuvant immune monotherapy in activating the body’s immune response is limited, often failing to achieve a good therapeutic effect. In the adjuvant stage, after the tumor has been removed, more attention should be paid to the side effects of the drugs when evaluating their efficacy. Research on immune checkpoint inhibitors is still in the exploratory stage. The phase I clinical study of immune checkpoint inhibitors reported by Marcq et al. was discontinued because of the high incidence of adverse gastrointestinal reactions (39). Radiotherapy is a critical component of conservative treatment. However, because there are many complex organs around the bladder, dysfunction of the bladder and surrounding organs occurs if the dose and scope of radiotherapy are not properly controlled. The initial dose was 40 Gy for the lymph nodes of the bladder and pelvic area, which increased to 46–54 and 64–66 Gy for the bladder alone and tumor bed, respectively (40). Even if the radiation dose is strictly controlled, it may lead to a range of adverse effects such as radiation cystitis and gastrointestinal dysfunctions.

With limited data, the role and risk assessment of surgical treatment in bladder cancer with multiple-organ invasion is far from clear; however, it demonstrates its feasibility. For patients who can tolerate surgery and intend to undergo surgery, surgical intervention has further shown a good benefit in tumor follow-up – prolonging the survival of bladder cancer patients with a short expected survival time under conservative treatment.

## 4 Conclusion

Our treatment process had some limitations, including incomplete clinical data and the lack of genetic or biomarker assays for drug screening. However, personal or practical factors cannot be ignored, and individualized strategies are required, considering the efficacy, toxicity, cost, availability of therapeutic options, and patient preferences. Minimally invasive surgery is a treatment modality for bladder cancer patients without distant metastasis but with pelvic multi-organ invasion. Surgeons should strictly control the indications for surgery, warn patients about the occurrence of related complications following surgical treatment, and choose an appropriate diversion procedure for feces and urine.

## Data availability statement

The original contributions presented in the study are included in the article/supplementary material. Further inquiries can be directed to the corresponding authors.

## Ethics statement

The studies involving humans were approved by Medical Ethics Committee of the First Affiliated Hospital of Jinan University. The studies were conducted in accordance with the local legislation and institutional requirements. The human samples used in this study were acquired from The specimens were surgically resected, and the follow-up specimens were sent to the Department of Pathology for immunohistochemistry and other procedures.Written informed consent was obtained from the individual(s) for the publication of any potentially identifiable images or data included in this article. Written informed consent for participation was not required from the participants or the participants’ legal guardians/next of kin in accordance with the national legislation and institutional requirements.

## Author contributions

CL, ZL, and YZ designed the study and edited and approved the manuscript. ZC and KD drafted the manuscript. KD and ZC were involved in the diagnosis, treatment, and follow-up of the patient and revision of the manuscript. LS, LQ, and XC collected and analyzed the data. All authors were involved in writing the manuscript. All authors contributed to the article and approved the submitted version.

## References

[1] Siegel RL Miller KD Fuchs HE Jemal A. Cancer statistics, 2022. CA Cancer J Clin (2022) 72:7–33. doi: 10.3322/caac.21708 35020204

[2] Lobo N Mount C Omar K Nair R Thurairaja R Khan MS. Landmarks in the treatment of muscle-invasive bladder cancer. Nat Rev Urol (2017) 14:565–74. doi: 10.1038/nrurol.2017.82 28675174

[3] Witjes JA Bruins HM Cathomas R Compérat EM Cowan NC Gakis G. European Association of Urology guidelines on muscle-invasive and metastatic bladder cancer: summary of the 2020 guidelines. Eur Urol (2021) 79:82–104. doi: 10.1016/j.eururo.2020.03.055 32360052

[4] Amin MB Comperat E Epstein JI True LD Hansel D Paner GP. The Genitourinary Pathology Society Update on classification and grading of flat and papillary urothelial neoplasia with new reporting recommendations and approach to lesions with mixed and early patterns of neoplasia. Adv Anat Pathol (2021) 28:179–95. doi: 10.1097/pap.0000000000000308 34128483

[5] Flaig TW Spiess PE Abern M Agarwal N Bangs R Boorjian SA. NCCN guidelines]® Insights: Bladder Cancer, version 2. 2022 J Natl Compr Canc Netw (2022) 20:866–78. doi: 10.6004/jnccn.2022.0041 35948037

[6] Seisen T Sun M Lipsitz SR Abdollah F Leow JJ Menon M. Comparative effectiveness of trimodal therapy versus radical cystectomy for localized muscle-invasive urothelial carcinoma of the bladder. Eur Urol (2017) 72:483–7. doi: 10.1016/j.eururo.2017.03.038 28412065

[7] Williams SB Shan Y Jazzar U Mehta HB Baillargeon JG Huo J. Comparing survival outcomes and costs associated with radical cystectomy and trimodal therapy for older adults with muscle-invasive bladder cancer. JAMA Surg (2018) 153:881–9. doi: 10.1001/jamasurg.2018.1680 PMC6584312 29955780

[8] García-Perdomo HA Montes-Cardona CE Guacheta M Castillo DF Reis LO. Muscle-invasive bladder cancer organ-preserving therapy: systematic review and meta-analysis. World J Urol (2018) 36:1997–2008. doi: 10.1007/s00345-018-2384-6 29943218

[9] Schuettfort VM Pradere B Quhal F Mostafaei H Laukhtina E Mori K. Incidence and outcome of salvage cystectomy after bladder sparing therapy for muscle invasive bladder cancer: a systematic review and meta-analysis. World J Urol (2021) 39:1757–68. doi: 10.1007/s00345-020-03436-0 PMC8217031 32995918

[10] Sloan FA Yashkin AP Akushevich I Inman BA. The cost to Medicare of bladder cancer care. Eur Urol Oncol (2020) 3:515–22. doi: 10.1016/j.euo.2019.01.015 31412015

[11] Clayman RV Kavoussi LR Figenshau RS Chandhoke PS Albala DM. Laparoscopic nephroureterectomy: initial clinical case report. J Laparoendosc Surg (1991) 1:343–9. doi: 10.1089/lps.1991.1.343 1838941

[12] Parra RO Andrus CH Jones JP Boullier JA. Laparoscopic cystectomy: initial report on a new treatment for the retained bladder. J Urol (1992) 148:1140–4. doi: 10.1016/s0022-5347(17)36843-x 1404624

[13] Petruţ B Coman RA Hârdo V Coste B Maghiar T. Laparoscopic radical cystectomy and nephroureterectomy en bloc resection with lomboaortic and pelvic lymph node dissection. Med Pharm Rep (2020) 93:390–5. doi: 10.15386/mpr-1626 PMC7664718 33225265

[14] Tanaka Y Okamura T Chaya R Nagai T Kobayashi D Kobayashi T. Outcomes and complications of simultaneous laparoscopic cystectomy and laparoscopic nephroureterectomy with umbilical reduced port surgery. Asian Pac J Cancer Prev (2018) 19:3495–500. doi: 10.31557/apjcp.2018.19.12.3495 PMC6428533 30583675

[15] Słojewski M Chłosta P Myślak M Herlinger G Dobroński P Kryst P. Single-session laparoscopic cystectomy and nephroureterectomy. Wideochir Inne Tech Maloinwazyjne (2013) 8:158–61. doi: 10.5114/wiitm.2011.31946 PMC3699764 23837100

[16] De Almeida R Qureshi Y Morawala A Merali N Iloabachie I Alaraimi B. Impact of 3D laparoscopic surgical training on performance in standard 2D laparoscopic simulation: a randomised prospective study. J Surg Simul (2018) 5:1–7. doi: 10.1102/2051-7726.2018.0001

[17] Poudel S Kurashima Y Watanabe Y Ebihara Y Tamoto E Murakami S. Impact of 3D in the training of basic laparoscopic skills and its transferability to 2D environment: a prospective randomized controlled trial. Surg Endosc (2017) 31:1111–8. doi: 10.1007/s00464-016-5074-8 27351662

[18] Cicione A Autorino R Breda A De Sio M Damiano R Fusco F. Three-dimensional vs standard laparoscopy: comparative assessment using a validated program for laparoscopic urologic skills. Urology (2013) 82:1444–50. doi: 10.1016/j.urology.2013.07.047 24094658

[19] Cologne KG Zehetner J Liwanag L Cash C Senagore AJ Lipham JC. Three-dimensional laparoscopy: does improved visualization decrease the learning curve among trainees in advanced procedures? Surg Laparosc Endosc Percutan Tech (2015) 25:321–3. doi: 10.1097/sle.0000000000000168 26053113

[20] Takagi T Kondo T Tachibana H Iizuka J Omae K Kobayashi H. Robot-assisted laparoscopic versus open partial nephrectomy in patients with chronic kidney disease: A propensity score-matched comparative analysis of surgical outcomes. Int J Urol (2017) 24:505–10. doi: 10.1111/iju.13363 28503809

[21] Mano R Goldberg H Stabholz Y Hazan D Margel D Kedar D. Urinary tract infections after urinary diversion-different occurrence patterns in patients with ileal conduit and orthotopic neobladder. Urology (2018) 116:87–92. doi: 10.1016/j.urology.2018.03.042 29626568

[22] Jordan BJ Lewis KC Matulewicz RS Kundu S. The timing and frequency of infectious complications after radical cystectomy: an opportunity for rescue antibiotic treatment. Urol Pract (2019) 6:34–9. doi: 10.1016/j.urpr.2018.01.003 37312339

[23] Parker WP Toussi A Tollefson MK Frank I Thompson RH Zaid HB. Risk factors and microbial distribution of urinary tract infections following radical cystectomy. Urology (2016) 94:96–101. doi: 10.1016/j.urology.2016.03.049 27125878

[24] Parker WP Tollefson MK Heins CN Hanson KT Habermann EB Zaid HB. Characterization of perioperative infection risk among patients undergoing radical cystectomy: results from the national surgical quality improvement program. Urol Oncol (2016) 34:532.e13–9. doi: 10.1016/j.urolonc.2016.07.001 27503783

[25] Magistro G Zimmermann L Bischoff R Westhofen T Grimm T Schlenker B. The natural course of urinalysis after urinary diversion. World J Urol (2021) 39:1559–67. doi: 10.1007/s00345-020-03355-0 32661555

[26] Kim KH Yoon HS Yoon H Chung WS Sim BS Lee DH. Febrile urinary tract infection after radical cystectomy and ileal neobladder in patients with bladder cancer. J Korean Med Sci (2016) 31:1100–4. doi: 10.3346/jkms.2016.31.7.1100 PMC4901003 27366009

[27] Calaway AC Jacob JM Tong Y Shumaker L Kitley W Boris RS. A prospective program to reduce the clinical incidence of Clostridium difficile colitis infection after cystectomy. J Urol (2019) 201:342–9. doi: 10.1016/j.juro.2018.09.030 30218764

[28] Furrer MA Schneider MP Burkhard FC Wuethrich PY. Incidence and perioperative risk factors for early acute kidney injury after radical cystectomy and urinary diversion. Urol Oncol (2018) 36:306.e17–23. doi: 10.1016/j.urolonc.2018.02.011 29550095

[29] Furrer MA Schneider MP Löffel LM Burkhard FC Wuethrich PY. Impact of intra-operative fluid and noradrenaline administration on early postoperative renal function after cystectomy and urinary diversion: a retrospective observational cohort study. Eur J Anaesthesiol (2018) 35:641–9. doi: 10.1097/eja.0000000000000808 29652680

[30] Shah SH Movassaghi K Skinner D Dalag L Miranda G Cai J. Ureteroenteric strictures after open radical cystectomy and urinary diversion: the University of Southern California experience. Urology (2015) 86:87–91. doi: 10.1016/j.urology.2015.03.014 25987494

[31] Studer UE Burkhard FC Schumacher M Kessler TM Thoeny H Fleischmann A. Twenty years experience with an ileal orthotopic low pressure bladder substitute—lessons to be learned. J Urol (2006) 176:161–6. doi: 10.1016/s0022-5347(06)00573-8 16753394

[32] Pantuck AJ Han KR Perrotti M Weiss RE Cummings KB. Ureteroenteric anastomosis in continent urinary diversion: long-term results and complications of direct versus nonrefluxing techniques. J Urol (2000) 163:450–5. doi: 10.1016/s0022-5347(05)67898-6 10647652

[33] Liu NW Hackney JT Gellhaus PT Monn MF Masterson TA Bihrle R. Incidence and risk factors of parastomal hernia in patients undergoing radical cystectomy and ileal conduit diversion. J Urol (2014) 191:1313–8. doi: 10.1016/j.juro.2013.11.104 24333109

[34] Heit JA Silverstein MD Mohr DN Petterson TM O’Fallon WM Melton LJ III. Risk factors for deep vein thrombosis and pulmonary embolism: a population-based case-control study. Arch Intern Med (2000) 160:809–15. doi: 10.1001/archinte.160.6.809 10737280

[35] Klaassen Z Arora K Goldberg H Chandrasekar T Wallis CJD Sayyid RK. Extended venous thromboembolism prophylaxis after radical cystectomy: a call for adherence to current guidelines. J Urol (2018) 199:906–14. doi: 10.1016/j.juro.2017.08.130 29113840

[36] Flaig TW Spiess PE Agarwal N Bangs R Boorjian SA Buyyounouski MK. Bladder cancer, version 3.2020, NCCN Clinical Practice Guidelines in Oncology. J Natl Compr Canc Netw (2020) 18:329–54. doi: 10.6004/jnccn.2020.0011 32135513

[37] De Santis M Bellmunt J Mead G Kerst JM Leahy M Maroto P. Randomized phase II/III trial assessing gemcitabine/carboplatin and methotrexate/carboplatin/vinblastine in patients with advanced urothelial cancer who are unfit for cisplatin-based chemotherapy: EORTC study 30986. J Clin Oncol (2012) 30:191–9. doi: 10.1200/jco.2011.37.3571 PMC3255563 22162575

[38] Galsky MD Pal SK Lin SW Ogale S Zivkovic M Simpson J. Real-world effectiveness of chemotherapy in elderly patients with metastatic bladder cancer in the United States. Bladder Cancer (2018) 4:227–38. doi: 10.3233/blc-170149 PMC5929305 29732393

[39] Marcq G Souhami L Cury FL Salimi A Aprikian A Tanguay S. Phase 1 trial of atezolizumab plus trimodal therapy in patients with localized muscle-invasive bladder cancer. Int J Radiat Oncol Biol Phys (2021) 110:738–41. doi: 10.1016/j.ijrobp.2020.12.033 33421558

[40] Nason GJ Ajib K Tan GH Kulkarni GS. Bladder-sparing treatment options in localized muscle-invasive bladder cancer. Expert Rev Anticancer Ther (2020) 20:179–88. doi: 10.1080/14737140.2020.1736565 32129122

